# Infection age as a predictor of epidemiological metrics for malaria

**DOI:** 10.1186/s12936-022-04134-5

**Published:** 2022-04-07

**Authors:** John M. Henry, Austin Carter, David L. Smith

**Affiliations:** 1 College of the Environment, University of Washington, 1492 NE Boat St., 98105 Seattle, USA; 2 Institute for Health Metrics and Evaluation, University of Washington, 3980 15th Ave. NE, 98195 Seattle, USA

**Keywords:** Malaria, Data, Infection age, Fever transmission efficiency

## Abstract

**Background:**

Accurate estimation of the burden of *Plasmodium falciparum* is essential for strategic planning for control and elimination. Due in part to the extreme heterogeneity in malaria exposure, immunity, other causes of disease, direct measurements of fever and disease attributable to malaria can be difficult. This can make a comparison of epidemiological metrics both within and between populations hard to interpret. An essential part of untangling this is an understanding of the complex time-course of malaria infections.

**Methods:**

Historic data from malariatherapy infections, in which individuals were intentionally infected with malaria parasites, were reexamined in aggregate. In this analysis, the age of each infection was examined as a potential predictor describing aggregate patterns across all infections. A series of piecewise linear and generalized linear regressions were performed to highlight the infection age-dependent patterns in both parasitaemia and gametocytaemia, and from parasitaemia and gametocytaemia to fever and transmission probabilities, respectively.

**Results:**

The observed duration of untreated patent infection was 130 days. As infections progressed, the fraction of infections subpatent by microscopy was seen to increase steadily. The time-averaged malaria infections had three distinct phases in parasitaemia: a growth phase for the first 6 days of patency, a rapid decline from day 6 to day 18, and a slowly declining chronic phase for the remaining duration of the infection. During the growth phase, parasite densities increased sharply to a peak. Densities sharply decline for a short period of time after the peak. During the chronic phase, infections declined steadily as infections age. gametocytaemia was strongly correlated with lagged asexual parasitaemia. Fever rates and transmission efficiency were strongly correlated with parasitaemia and gametocytaemia. The comparison between raw data and prediction from the age of infection has good qualitative agreement across all quantities of interest for predicting averaged effects.

**Conclusion:**

The age of infection was established as a potentially useful covariate for malaria epidemiology. Infection age can be estimated given a history of exposure, and accounting for exposure history may potentially provide a new way to estimate malaria-attributable fever rates, transmission efficiency, and patent fraction in immunologically naïve individuals such as children and people in low-transmission regions. These data were collected from American adults with neurosyphilis, so there are reasons to be cautious about extending the quantitative results reported here to general populations in malaria-endemic regions. Understanding how immune responses modify these statistical relationships given past exposure is key for being able to apply these results more broadly.

**Supplementary Information:**

The online version contains supplementary material available at 10.1186/s12936-022-04134-5.

## Background

Despite great progress in recent decades, malaria from *Plasmodium falciparum* infection continues to claim approximately 435 thousand lives each year [1, 2]. Deaths represent only part of the overall burden, as an estimated 194 million cases occurred in 2017 alone [1, 2] despite the existence of cures and preventative measures. Malaria therefore represents a major source of avertible disease burden, with a major challenge being how to efficiently allocate resources to people in need. Assessments of potential targeted interventions to efficiently reduce prevalence depend on detailed knowledge of the epidemiology in the region of interest gained from local research studies.

Epidemiological surveys of malaria typically report some combination of asexual parasite counts, clinical incidence, prevalence, and rates of fever to compare age-specific patterns of disease and transmission among populations [3, 4]. However these multifaceted data paint a complex picture, with patterns in routine clinical surveillance data that are difficult to interpret due to ambiguity in the causes of observed trends. Therefore it is important to characterize patterns which appear in first infections in order to determine how they may be altered with different levels of past exposure. To this end, this study aims to establish time since infection as a predictor of patent infection probability by microscopy, asexual parasite densities, gametocyte densities, fever probabilities, and transmission efficiency. This relationship can be leveraged in future modelling studies which can track theoretical estimates of infection age given rates of exposure, and incorporate the effects of immunity to see how these baseline patterns in individuals may be altered to lead to the population level patterns seen in data.

Previous research on the time course of infection has described the highly volatile trajectories of parasitaemia in a single individual over time, whose counts can jump orders of magnitude over the course of a day [5, 6]. Studies focused on infection durations have estimated that the average time to the last observed patent infection ranges from around 100 days to over 1000 days [7–9], with at least one confirmed infection persisting for over a decade [10]. Patterns in the spikes and troughs of parasite counts have been investigated for evidence of mechanisms driving patterns such as VAR gene switching in parasite densities [9] or blood cell age preferences of the parasites [11]. Insight derived from these studies are valuable, but it is difficult to interpret these mechanisms’ role in the efficacy of a particular intervention, or to scale to the impact on country or continent level estimates of burden. Asexual parasitaemia is a standard covariate for estimating the malaria attributable fraction of fever [3] and the heterogeneous relationship between gametocytaemia and transmission efficiency [12–14] at the population level, but there remain lingering issues of identifiability regarding the impact of immunity.

**Fig. 1:**
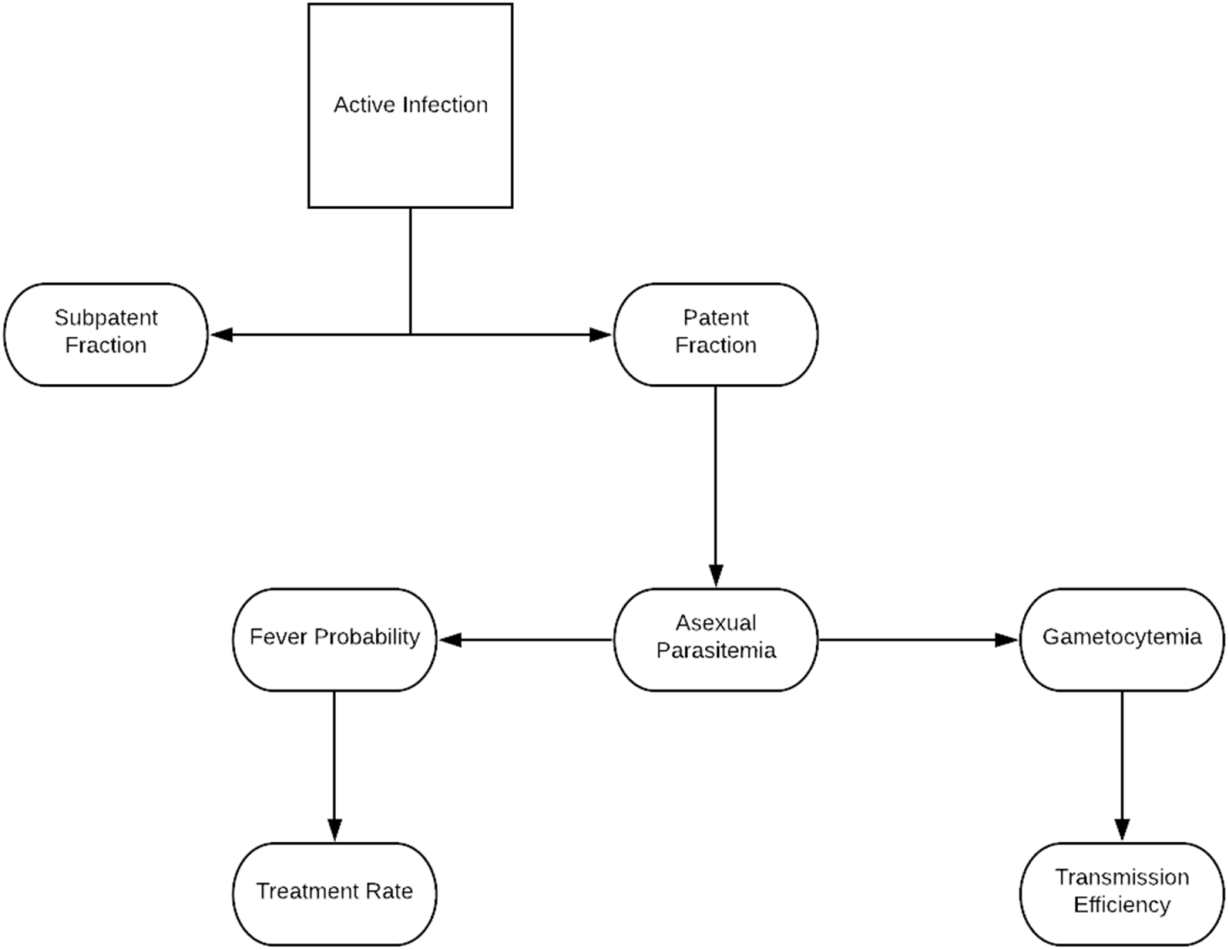
Schematic breakdown of infections. Given infections are active, some fraction will be subpatent (i.e., undetectable by light microscopy) and the rest will be patent. Given the parasitaemia is patent, we can estimate the parasitaemia which will inform fever probability today and gametocytaemia roughly 9 days in the future. Fever rates presumably are correlated with treatment rates as symptomatic individuals are much more likely to seek treatment, and gametocytaemia is positively correlated with the transmission efficiency per mosquito bite

Due in part to widely varying histories of past exposure among individuals in a population, observational studies of the relationship between quantities such as prevalence, malaria attributable fever, and per capita transmission rate may be misleading if they do not take into account the heterogeneous effects of immunity. Innate immune responses may differ in individuals depending on exposure, age, and other possible underlying conditions, and adaptive immune responses may differ based on an individual’s history of past exposure. The effects of immunity limit an infection, reducing parasite population growth and eventually clearing infections. The impact of adaptive immunity on subsequent infections can further be decomposed into five categorical effects: pre-erythrocytic immunity, anti-(asexual) parasitic immunity, parasite tolerance, anti-gametocytic immunity, and transmission blocking immunity. Pre-erythrocytic immunity slows or prevents the establishment of an infection before or in the liver. Anti-parasitic immunity acts to reduce the blood stage parasitaemia, which is correlated with disease. Parasite tolerance modifies the relationship between parasitaemia and disease; it is measured as a reduction in the likelihood of fever and other clinical symptoms for a given parasitaemia. Anti-gametocytic immunity reduces gametocyte densities. Gametocytes infect mosquitoes, and their densities are correlated with transmission efficiency. Transmission blocking immunity reduces transmission efficiency for a given gametocytaemia, analogous to parasite tolerance [13]. This is mediated through immune effectors which target gametocyte-specific antigens, compounding the impact of antiparasite immunity which reduces the number of asexual parasites which produce gametocytes. Malaria epidemiology and immunity can therefore be understood as a set of cascading consequences of parasitaemia (Fig. 1). As these different modalities will impact fever, patency, and transmission rates, a direct translation from parasitaemia or prevalence to other epidemiological measures without previous exposure taken into account may be difficult to establish at best and lead to spurious patterns at worst.

Understanding how *P. falciparum* infections develop in the absence of acquired immunity is thus key to understanding and interpreting malaria data in which immunity has modified baseline patterns. Baseline data are difficult to obtain in malaria-endemic areas, but data describing some malaria infections in non-immune individuals is available from the historical data describing carefully monitored, deliberate malaria infections used to induce a fever to treat neurosyphilis, called malariatherapy [5, 15–19]. The malariatherapy data used in this study was initiated through either intravenous injection with asexual parasites directly or mosquito bite with sporozoites. They have previously been used to study the duration of single infections [20]. This is difficult to estimate from longitudinal studies due to the relatively common occurrence of superinfection, in which individuals are infected with multiple cohorts of parasites simultaneously, and unknown past exposure. Some recent studies have used genetic data to follow individual infections and estimate the multiplicity of infection [21, 22] but such studies can only detect genetically distinct strains sporadically, and infections with the highest densities at the time of measurement mask the presence of lower density infections. Some malariatherapy patients were infected several times, and their records have been used to study the effects of adaptive immunity by comparing the difference between homologous and heterologous challenge; effects of immunity to homologous challenge appear to be present after one or two infections, but it may take more exposure for strain transcending immunity to occur [23].

In this study, focus was placed on population-averaged patterns in parasite densities over the time course of the infection in individuals with no prior exposure as a reference for uncomplicated malaria with no effects of previously acquired immunity in adults using a sample from the malariatherapy patient data. Statistical relationships were established between average parasitaemia and epidemiological measures such as fever rates and transmission efficiency in relation to recent exposure in immunologically naïve individuals (Fig. 1). Given an infection has not been cleared, it can either be patent or subpatent; given patency, individuals will have some measurable asexual parasitaemia. This parasitaemia is used as a measure of severity of disease, and therefore risk of symptoms such as fever. Asexual parasites also produce gametocytes after some maturation period, previously estimated to be 9–12 days [24, 25], which persist with a short half-life [26]. In turn, gametocytaemia is used as a predictor for transmission efficiency [13, 14]. This framework was used as a lens through which to statistically view the aggregate data, demonstrating that the age of an infection can be used as a potentially powerful surrogate for estimating these hard to measure and dynamic quantities.

## Methods

The data set consists of 316 adult patient records, 258 males and 58 females of unreported ages, between 1941 and 1954 in the American south. The participants were patients with neurosyphilis being treated with malaria parasites, which were intended to induce a fever and effective immune response against spirochetes to mitigate outcomes of neurosyphilis. The infections were initiated through injection with either sporozoites to induce a liver infection or merozoites to directly induce blood stage infection. Results were aggregated across both inoculation methods, as previous results have shown little difference in measured quantities in this study between the two groups or by dose administered beyond the prepatent period, which was not investigated here [27, 28]. Infection age was counted from the first day of patency of an infection. Little clinical difference in outcomes aside from latency before first measurement was noticed, so both types of exposure were included here. Once patent by microscopy, daily measurements were taken of asexual and sexual stage parasites, and body temperature if there was an apparent fever. If symptoms of malaria became severe or if parasite densities were too high, patients were given treatment inadequate to cure malaria but sufficient to reduce parasitaemia. Treatment was not standardized and included a range of 28 different drug regimens. When there were detectable gametocytes in the blood, mosquito feedings were performed to determine the transmission efficiency from human to mosquito. Once the infection had been subpatent for some time, full treatment was given to clear the parasites entirely. In a subset of patients, secondary infections were initiated through either homologous or heterologous challenge. No deaths were reported.

For the estimate of duration of patent infection, we excluded treated cases (treated n = 189). For all other estimation, we included infections that were treated until the day they were first treated, where they were truncated (n = 299). On any day that an individual had patent gametocytaemia, a mosquito feeding was performed and after the estimated extrinsic incubation period the mosquitoes were dissected to determine the fraction which became sporozoite positive (n = 2029 observations). The subsequent infection challenges that occurred in some patients were excluded, as we were interested in the course of first infections.

The primary features of interest included infection duration, patent fraction over time, asexual parasitaemia over time, gametocytaemia over time, fever risk associated with parasitaemia, and transmission efficiency associated with gametocytaemia. The fits of the relationships between all the observable quantities are summarized in Table 1. For patency, asexual parasitaemia, and gametocytaemia, piecewise linear or generalized linear fits were performed and summarized. The distributions of asexual parasitaemia and gametocytaemia were also represented in violin plots aggregated by month to show general trends. Daily means and variances appeared to have a relationship, so a power law was fit. Logistic regressions were performed to translate daily average parasitaemia to fever risk, and smoothed gametocytaemia to transmission efficiency. The degree of zero inflation in the transmission efficiency and the beta-fitted histograms of transmission efficiency across binned levels of gametocytaemia were also plotted to emphasize the overdispersion of the relationship. Code for the analysis is included in additional file 1.

**Table 1:** Fitted relationships between infection age and quantities of interest

Quantity of interest	Fitted equation of Kernel
Patent Fraction, D($\alpha$)	$${\left\{ \begin{array}{ll} 1 &{} \text {if}\ \alpha \le 6 \\ 1.12 - .02 \ \alpha &{} \text {if}\ 6 < \alpha \le 18 \\ \left( 1+e^{-1.52+0.0151 \alpha }\right) ^{-1} &{} \text {if}\ \alpha >18 \end{array}\right. }$$
$\log _{10}$ Asexual parasitaemia, P($\alpha$)	$${\left\{ \begin{array}{ll} 0, &{} \text {if}\ \alpha \le 0 \\ 3.10 + .278 \ \alpha , &{} \text {if}\ 0< \alpha \le 6 \\ 5.12 - .0743 \ \alpha , &{} \text {if}\ 6 < \alpha \le 18 \\ 3.85 - .00843 \ \alpha , &{} \text {if}\ \alpha >18 \end{array}\right. }$$
Fever Probability, F(P($\alpha$))	$\frac{.859 e^{3.45 P(\alpha )}}{58200 + e^{3.45 P(\alpha )}}$
$\log _{10}$ gametocytaemia, G($L_9 P(\alpha )$)	$-0.684+.892L_9 P(\alpha ), L_9 P(\alpha ) = P(\alpha -9)$
Transmission Efficiency, c(G($\alpha$))	$\frac{.683 e^{2.14 G(\alpha )}}{131 + e^{2.14 G(\alpha )}}$

Patent fraction consisted of two piecewise-linear fits and a generalized linear fit at each of the transitions mentioned in the text. Log parasitaemia likewise is piecewise linear. Fever probability and transmission efficiency are logistic functions of their predictors, which are linear functions of infection age

## Results

**Fig. 2:**
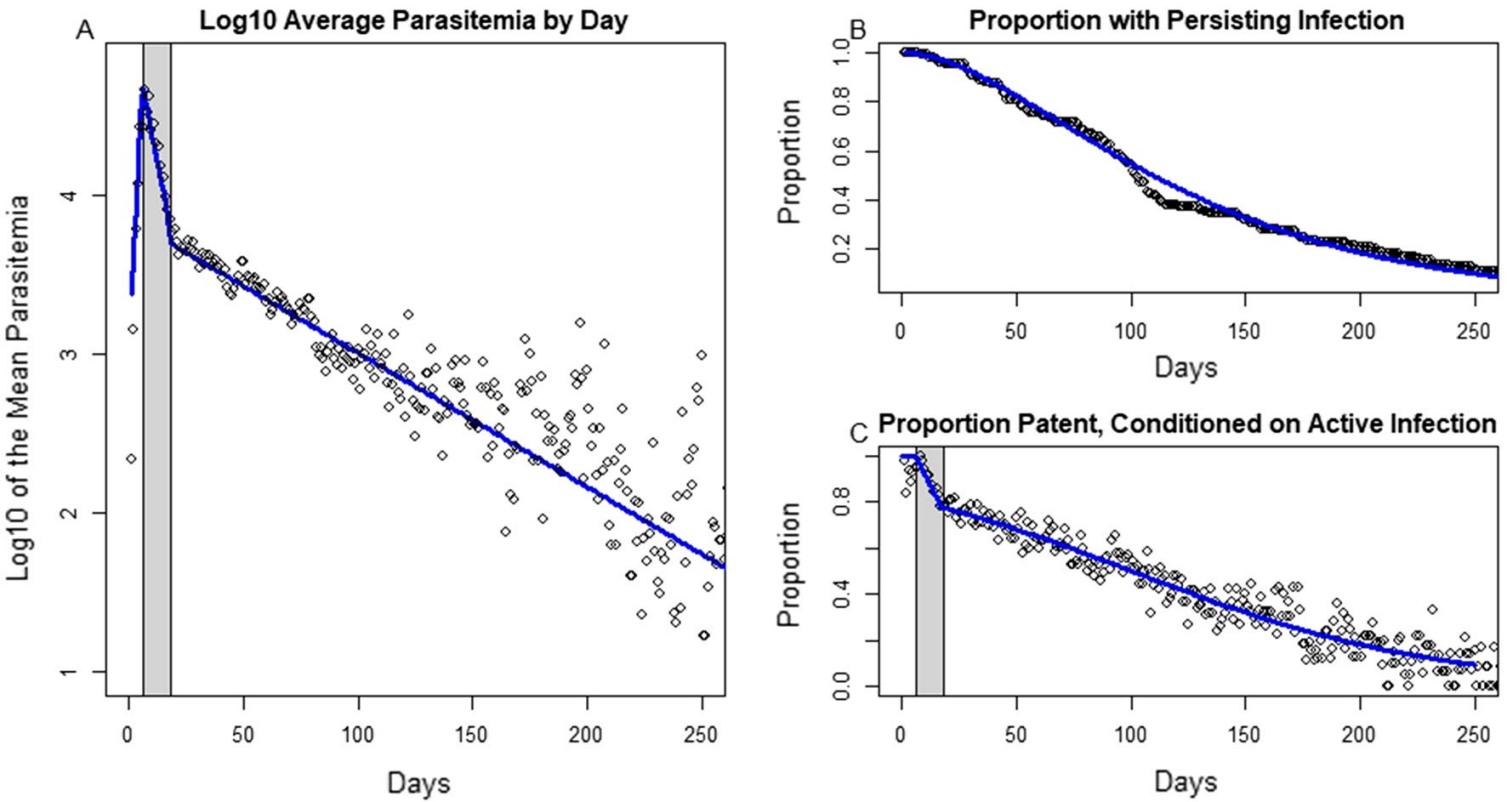
Plots of parasitaemia, duration, and patency. **A** Shows a scatter plot of the log10 of the daily average parastemia among patent infections conditioned on continued infection. The blue lines are a three piecewise linear fit in three parts, with a gray shaded region representing the middle of the three. **B** Shows a plot of the empirical survival function of infections that had positive measures of parasitaemia on or after that day of infection. The blue curve represents the best fit gamma survival curve, that is the complement of the corresponding gamma CDF. Finally **C** is a plot of the proportion patent conditioned on continued infection. The gray shaded region is the same highlighted in panel A, showing that during the initial growth phase nearly all infections remain patent; then patency drops about 20 percent over a short time, then it slowly decays to around 10 percent by day 250

The average duration of the infection, restricted to the subset of patients who were untreated during the entire infection (n = 110), was estimated to be 130 days (Fig. 2b). Here the duration was defined as the age of infection on the last day with patent asexual parasitaemia by microscopy, which was followed by a sequence of parasite negative observations and the cessation of measurement. We compared exponential, gamma, weibull, and lognormal survival curves, with delta AIC (23.3, 0, 1.7, 6.8, respectively) and delta BIC (20.6, 0, 1.7, 6.8, respectively) confirming exponential as a poorest fit and gamma and weibull as being the best candidates. The plotted blue curve is the gamma survival curve, with shape parameter 2.058 and rate parameter 0.015 per day.

For the analyses that follow all infections were included, in particular observations of all infections until they were ended by treatment. This allowed a reduction of the bias introduced by removing all infections with higher parasitaemia which tended to be treated at higher rates.

Asexual parasite densities followed a three-phase pattern (Fig. 2). For the first six days of a patent infection, parasite densities had increased geometrically. This was followed by a sharp decline between days 6–20. After that, here referred to as the chronic phase, parasite densities and the fraction patent on a given day slows to a shallower nearly linear trend in time. Among patent infections, the log10 transformed daily average declined linearly (Fig. 2a). In the chronic phase, infections sometimes had subpatent periods before spikes in parasitaemia occurred again. The proportion of peristent infections which were patent on a given day declined steadily (Fig. 2c). Older infections tend to spend a signficant fraction of time at submicroscopic densities in the blood with occasional bouts above that theshold of detectability, somewhere around 88 parasites per cmm of blood [29]. The increasing variance in points around the fitted average was due in part to the sample size in the daily averages decreasing as individuals either receive treatment or recover. Additional variation had occurred to a smaller fraction of those with persisting infection remaining patent, so many of the later plotted points are representative of a small number of individuals with late spikes in parasitaemia.

**Fig. 3:**
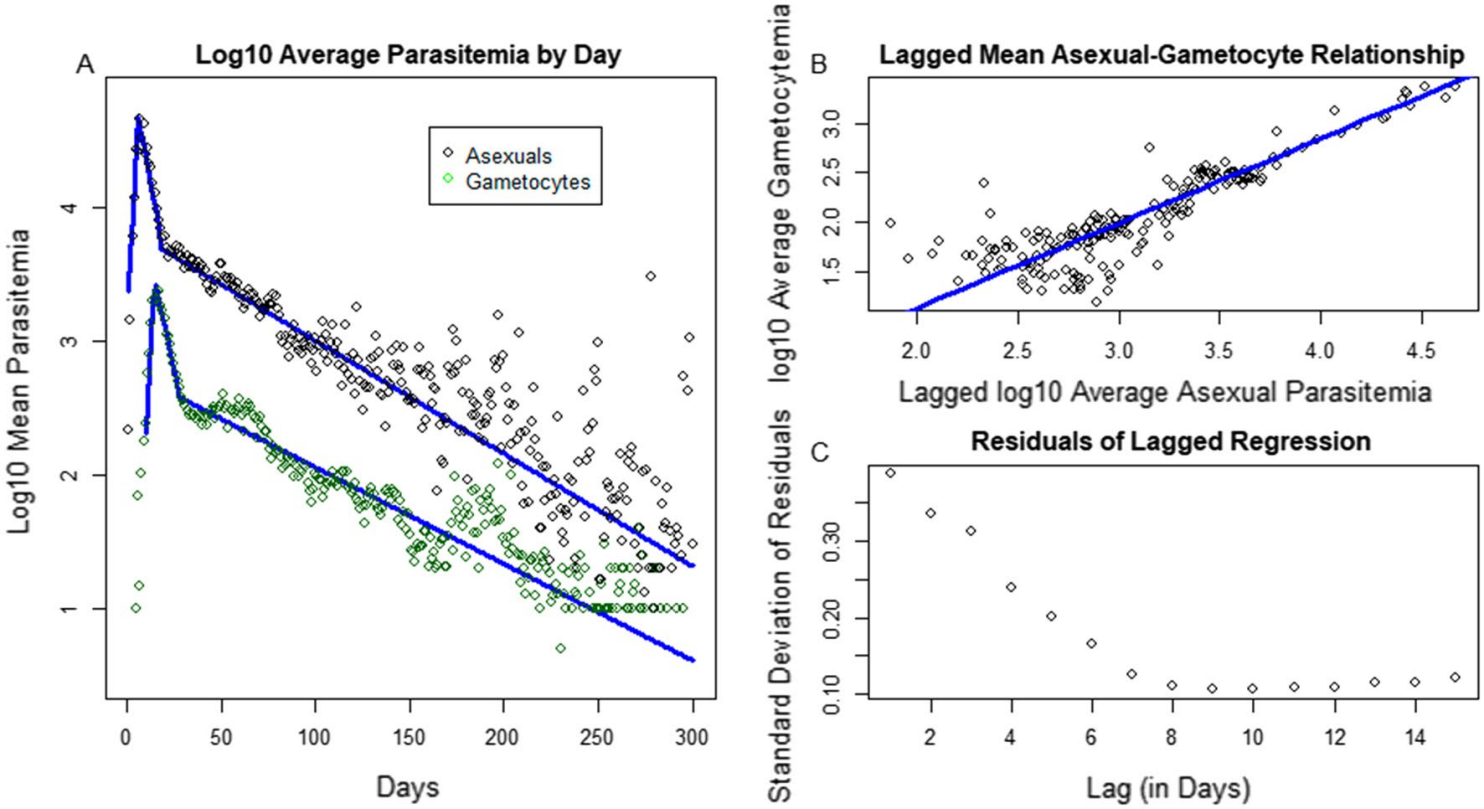
**A** Shows two time series of points, the top representing the log10 of the daily mean parasitaemia on a given day across all continued patent infections as in Fig. 2A and the bottom green time series represents the corresponding log10 daily mean gametocytaemia. A fit to predict gametocytaemia from lagged parasitaemia was performed across many lags, and the optimal lag was determined by the minimum of the standard deviation of the lagged residuals shown in **C**. The linear fit at the optimal lag of 9 days in presented in panel **B**

A strong relationship between asexual parasitaemia and gametocytaemia became apparent. The log10 of the average parasitaemia and gametocytaemia appeared to be shifted and scaled versions of one another (Fig. 3a). Lagged average parasitaemia as a linear predictor of gametocytaemia was therefore explored. The optimal lag was determined to be 9 days, which minimized the standard deviation of the residuals (see the trough of Fig. 3c). The flat nature of the standard deviation as a function of the lag around days 8–12 suggests that other lags may be nearly as good of fits, which is consistent with the estimated maturation period of gametocytes [24, 25]. Gametocyte densities in the chronic phase had also declined linearly (Fig 3b). After accounting for the lag, gametocyte densities were approximately 10-fold lower than parasite densities.

**Fig. 4:**
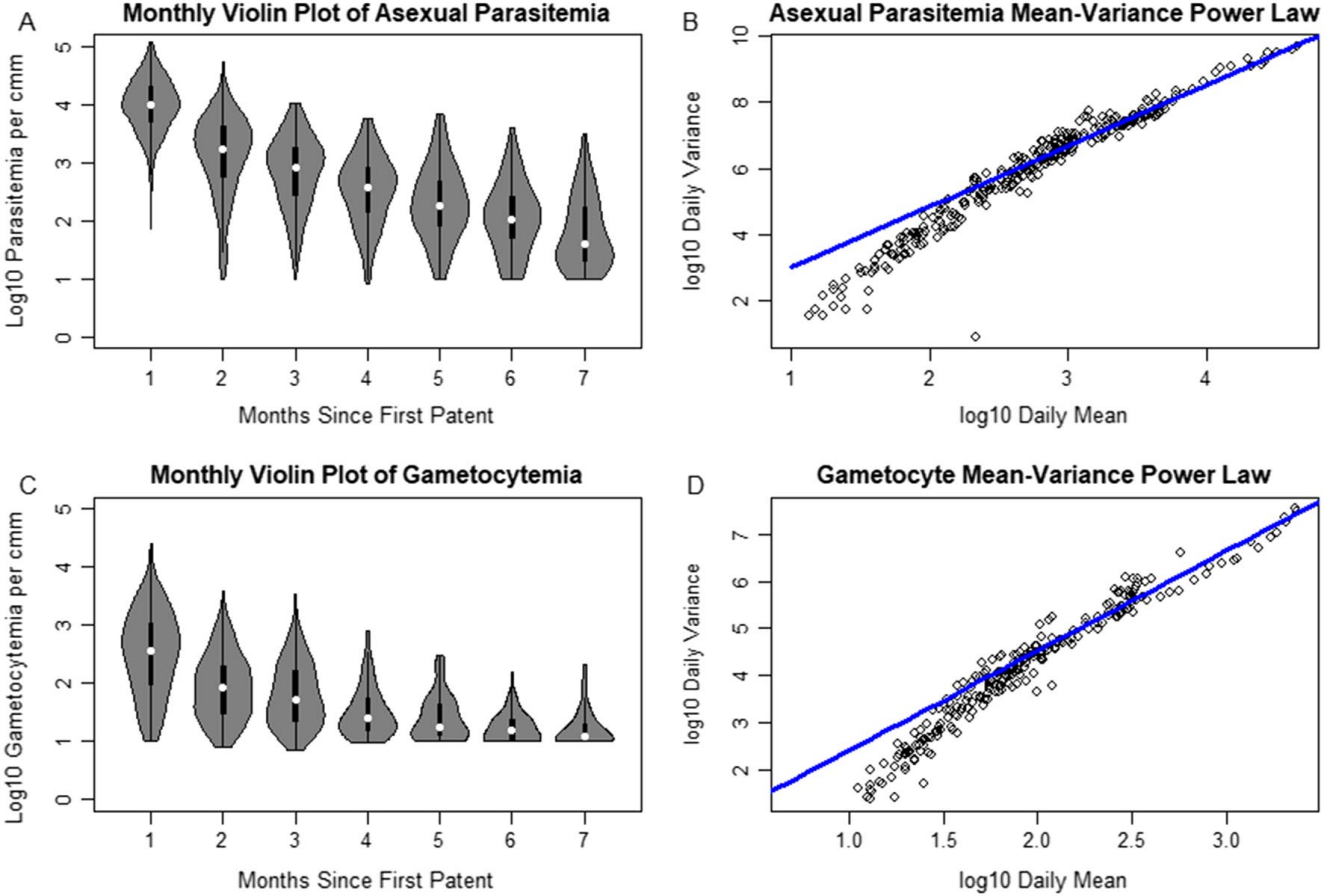
**A**, **C** Represent violin plots of the distributions across individuals of respectively the log10 daily parasitaemia and log10 daily gametocytaemia across all individuals, aggregated across 30 day periods for compactness. Despite being log transformed, the distribution shapes appear to shift down with infection age but maintain the same general shape, suggesting a power-law relationship between the mean and variance of parasitaemia. Power laws are then fit to log10 transformed daily mean and variances in **B** and **D**, restricted to those above the estimated sensitivity of light microscopy (88 parasites per cmm blood)

In addition to patterns in average asexual parasitaemia and gametocytaemia, patterns in the distribution across all individuals on a given day were investigated (Fig. 4). Monthly violin plots of the asexual parasitaemia and gametocytaemia after log-transformation appeared to maintain their shape while shifting down as infections aged. A power law relationship between mean and variance of both asexual parasitaemia and gametocytaemia for patent infections was quantified (Fig 4b, d). Power laws are often known to exist in higher density regions [30], although a decrease in variance may occur in measurements near the threshold of detectability by light microscopy as lower measurements are likely to be recorded as subpatent and therefore not included in daily measurements.

**Fig. 5:**
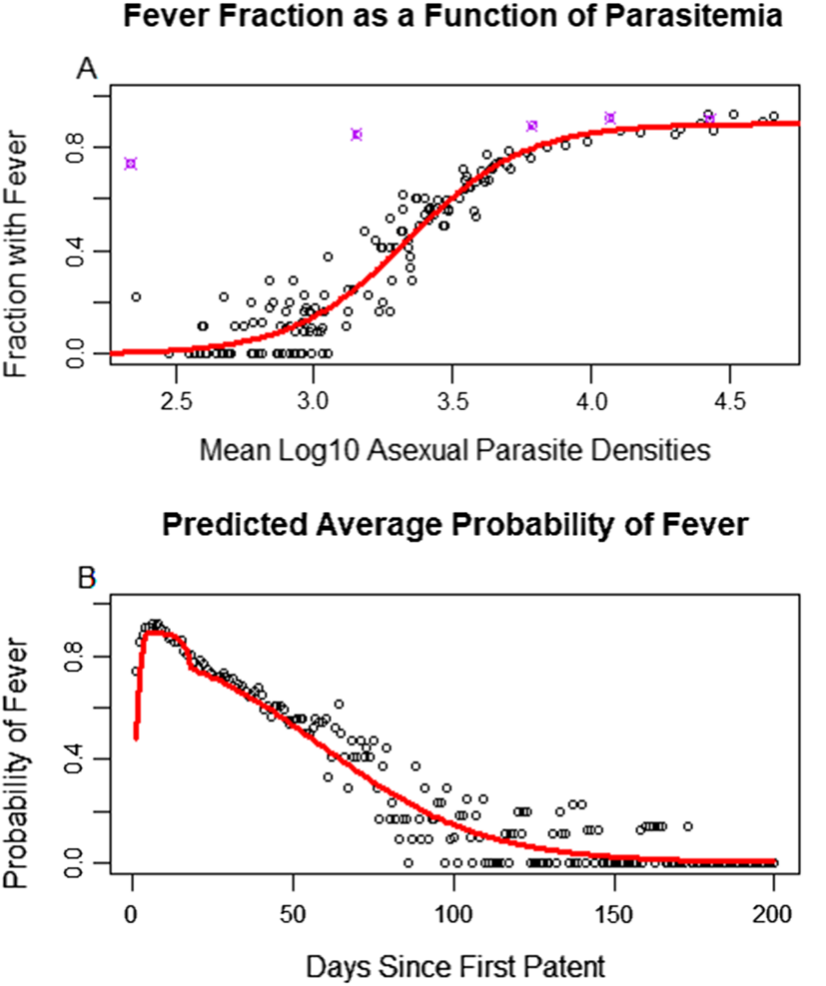
**A** Shows a logistic fit of the log10 of daily mean asexual parasitaemia to the fraction of individuals with objective fever. The five points in purple above the logistic curve are the first 5 days of infection, suggesting that many febrile individuals got primary fevers before high density infections. **B** Shows a time series of the daily fraction of individuals with active infections who have fever. The red curve represents the transformed piecewise fit of parasitaemia transformed through the fitted logistic curve

Log10 daily measurements of parasitaemia were then fit as a predictor of the proportion of individuals with fever, conditioned on patent parasitaemia. The best fit was a sigmoid function from the GLM with logit link (Fig. 5). There was a different relationship between parasite densities and fever in the first five days of patency compared to the rest of the infection. Note the five purple points laying above the sigmoid, with the leftmost two in particular being strong outliers. These points represent the first five days of infection, with the days being ordered from left to right. This would imply parasitaemia is a poor predictor in the first few days of infection, and in particular fever may come days before high parasitaemia. Unsurprisingly, fever was also a function of the age of the infection. The fit appears to follow the data very well, though underestimates the first few days of infection as expected.

**Fig. 6:**
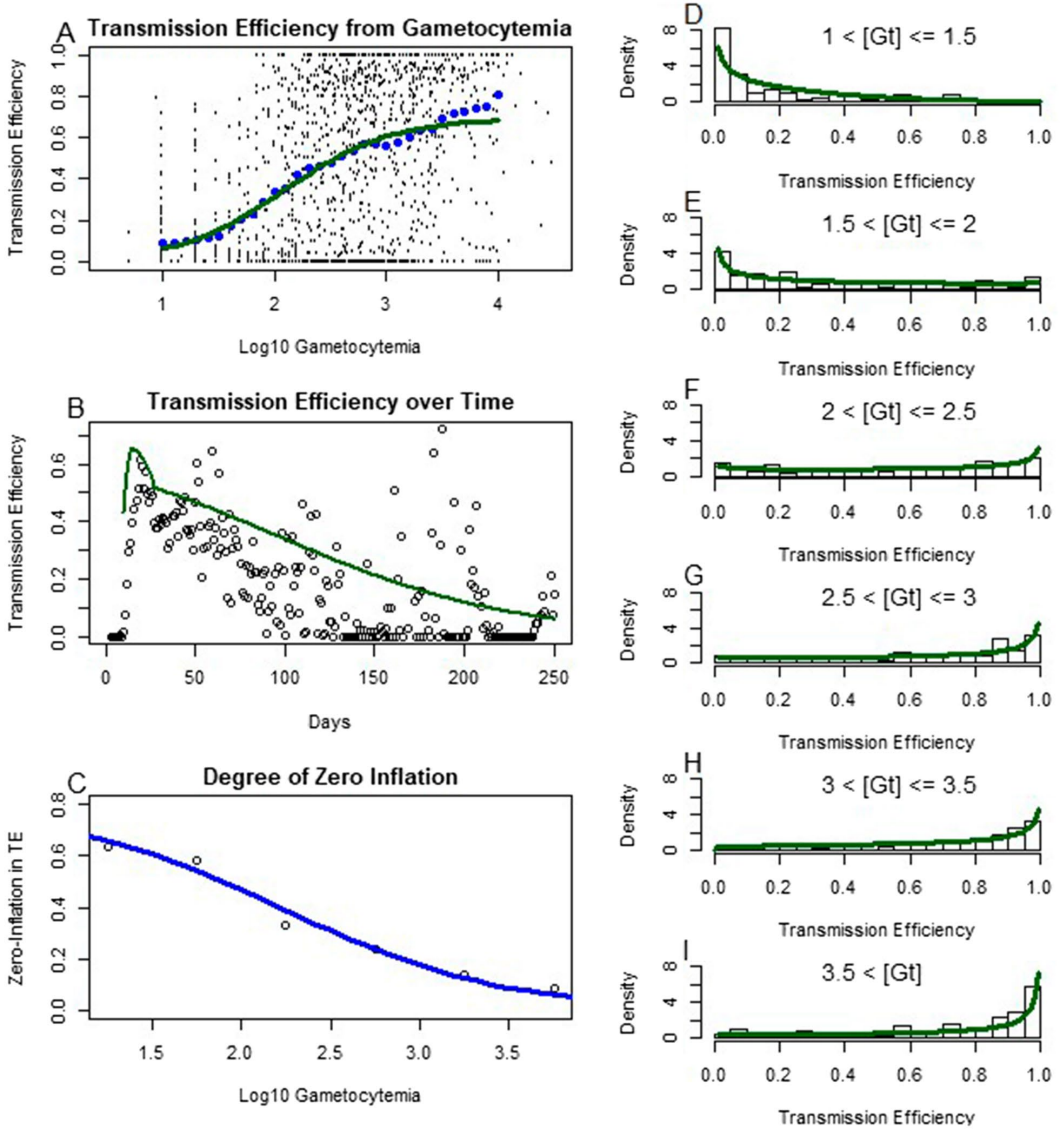
The black points in **A** represent the transmission efficiency, measured through the fraction of fed mosquitoes who developed sporozoites, against the log10 gametocytaemia measured on that day for each individual on every day they were gametocyte positive. Treated infections were not included in this analysis. Blue points are a rolling average of transmission efficiency as a function of log10 gametocytaemia, and the green curve is a sigmoid curve fit to these points with weights given by the number of points included in their rolling average. **B** Shows the daily average transmission efficency as a function of infection age, with the green curve representing the measured log10 daily average gametocytaemia composed with the sigmoid fit in **A**, showing good qualitative agreement. As the relationship between gametocytaemia and transmission efficiency is highly heterogeneous and zero-inflated, zero-inflated beta distributions were fit to binned values of gametocytaemia to quantify this. **C** shows the degree of zero inflation, that is the fraction of mosquito feedings resulting in no infections at all, as a function of log10 gametocytaemia. The blue sigmoid curve was fit to these points, showing the apparent zero-inflation decreases with increasing gametocytaemia. Finally, **D**–**I** are histograms of transmission efficiency for a given range of gametocytaemia conditioned on nonzero measurements, with beta distributions fit to each. Despite the high degree of heterogeneity, density can be seen to aggregate to the right with increasing gametocytaemia

Finally the relationship between gametocytaemia and transmission efficiency was quantified. Transmission efficiency was measured as the fraction of mosquitoes that developed sporozoites after feeding on individuals with patent gametocytaemia. The relationship between gametocytaemia and transmission efficiency was consistent but noisy. To fit the data, the data generating process was modeled as a mixture process, a zero-inflated beta-binomial distribution. Smoothing individual measurements across log10 gametocytaemia by averaging over measurements with similar gametocytaemia resulted in the blue points in Fig. 6a. A logistic regression was performed on the blue points, with weights proportional to the number of measurements used in the average. Analogous to Figs. 5b, 6b shows the log linear fit of gametocytaemia filtered through the sigmoid in green.

The beta-binomial interpretation had allowed for quantification of overdispersion, with zero-inflation added due to the large abundance of zeroes. Interestingly, the amount of zero inflation had appeared to decrease with increasing gametocytaemia, as shown in Fig. 6c. Conditioning on a nonzero number of mosquitoes counted, the histograms were plotted in binned gametocytemia with beta distributions fit in Fig. 6d–i. As expected, increasing gametocytaemia had shifted likelihood to higher transmission efficiency but with a large amount of variability. Therefore even highly gametocytemic patients often infected less than expected mosquitoes, or even none at all, during a particular feeding. This could have been partially explained by a result of fairly small samples of mosquitoes feeding successfully per patient per day, though another mechanism cannot be ruled out such as differences in sex ratios of gametocytes or strain-specific differences that are accentuated in transmission more than disease states [31].

## Discussion

It was shown that asexual parasite densities were strongly predicted by the age of infection, the relationship between parasite densities and fever and gametocytaemia and the relationship between gametocytaemia and infectiousness were quantified. In particular, estimation of the expected asexual parasitaemia was determined as a function of infection age, from which every other quantity can be estimated. The diagram in Fig. 1 shows pathways through intermediate quantities to translate from infection age to outcomes of interest. As there is also have a relationship between the mean and variance of asexual parasitaemia and gametocytaemia, fitting a family of distributions allows for estimation of full distributions of fever rates and transmission efficiencies as well. Therefore this represents a potentially powerful framework for future estimation.

This analysis suggests it may be possible to translate knowledge of a history of exposure to epidemiologically important quantities which are strongly correlated to the age of the infection if acquired immunity can also be estimated. Averaging these conditional rates on the probability an individual has had an infection for some duration across all present infection ages weighted by the fraction of the population who has had an infection for that duration gives expected population-level metrics in a given transmission setting. The basic idea follows the law of the unconscious statistician. If for example one were to estimate some observable *X* which depends on the concentration of the pathogen *p*, the following could be computed:

$$\begin{aligned} E[X_p] = \int _0^{\infty } P(p = p_0) E[X_{p_0}|p_0] dp_0. \end{aligned}$$

Examples of such *X* would include fever rates and transmission efficiency, as each would reasonably increase on average with pathogen density. However, this would require knowledge of the probability that an individual in the population has a pathogen concentration of *p*. This is rarely known and difficult to measure directly, as surveys often only measure incident cases which tend to have higher parasitaemia, and detection rates themselves depend on pathogen densities. However due to the observation that malaria appears to exhibit strong average parasite density patterns with respect to the age of an infection, it would be possible to parameterize *p* (and therefore X) through the infection age $\alpha$. This would give

$$\begin{aligned} E[X_{p(\alpha )}] = \int _0^\infty P(\alpha = \alpha _0) E[X_{p(\alpha _0)}|\alpha _0] d\alpha _0. \end{aligned}$$

One advantage to this formulation would be to allow one to estimate the probability of having an infection of age $\alpha$ through the use of standard age of infection dynamics given estimates of exposure [32]. The resulting distribution could give a reasonable estimate on the unknown probability given patterns in rates of exposure, allowing one to compute the desired expectation.

Past work on malaria attributable fever [3], which estimated malaria attributable fraction of total fever based on parasitological survey data of children, is analogous to the parasitaemia-to-fever risk regression done here. This interpretation would allow for the logistic regression to assign a probability to any child with parasite density measurements and a fever to determine how likely it is that the fever they have is attributable to malaria. The data was restricted to relatively young children, so the effects of adaptive immunity could be largely ignored as they were here. However to apply those results to an entire population, the regression would need to be reworked across measurements of all age groups as their past exposure and developed immunity will modify parasite densities and fever tolerance. Additionally, exposure history may vary dramatically from location to location, so the method would require an enormous amount of data and each location with its unique history of exposure could have a very different estimate from even other locations with the same current day prevalence. The analysis which was presented here shows that if one is instead able to estimate how long ago individuals were most recently infected based on a history of exposure from historical prevalence data, it would be possible to estimate a malaria attributable fraction of fever in the absence of parasite density surveys given a reasonable model of immunity.

Although parasitaemia appears to be a very good predictor of fever after the first week or so of patent parasitaemia, the fever rate was seen to be consistently higher than predicted by parasitaemia in the early days of patency. This would suggest the notion of a difference between primary fever, caused at the beginning of an infection, and a secondary fever, correlated strongly with parasitaemia and occurring later in the infection. This could be a consequence of the inflammatory cascade early in infection which is subsequently tempered by anti-inflammatory responses as the immune response has matured. An uncontrolled early inflammatory response is often found in severe cases of malaria [33, 34], so mortality in cases may be closely correlated with this early stage of infection.

Analogous to the fever and asexual parasitaemia relationship, the infection reservoir and its impact on estimation of the human-to-mosquito transmission potential in environments with seasonal transmission may be largely impacted by the additional heterogeneity presented here. In addition to the overdispersion shown in the translation from gametocytaemia to transmission efficiency, the infection-age dependent patterns of infectivity may be leveraged to improve our understanding of which locations may be a “source” or “sink” for malaria transmission, and how a location may switch from one to another based on the history of recent exposure and the age group of individuals in question [35, 36].

The fit for transmission efficiency as a function of gametocyte densities had appeared to be consistently above the data. This was due to a technical difference in the fitting procedure compared to fever. While fever was predicted from daily average parasitaemia, transmission efficiency was predicted from a function of the gametocytaemia measurements and not to the averaged time series data directly. Combined with the fact that most of the gametocytaemia measurements in the time series are from the top half the fitted sigmoid and therefore filtered through a concave function, Jensen’s inequality would guarantee that plugging in the mean to the function rather than taking an average of the data filtered through the sigmoid is expected to be an overestimate. However either through computing the conditional expectation mentioned above or simulating draws of gametocytaemia on a given day then converting through the sigmoid function, this problem would be avoided completely.

The power law relationships demonstrated between the mean and the variance, which suggests evidence of the well-known Taylor’s Law from ecology [37], could be used for practical computations here. This relationship is often seen between the means and variances of populations across different spatial regions, but would apply here as each human host can be imagined as an independent habitat for the parasite populations. One of the significant advantages of it is it would allow one to use the relatively simple pattern in mean parasitaemia over time to obtain a similar pattern in variance over time, and therefore parametrically could describe a wide class of two-parameter distributions for parasitaemia as a function of infection age through moment matching. This allows for the propagation of uncertainty of estimates through the relationships in a way which would circumvent the issues of Jensen’s inequality mentioned above.

It is necessary to highlight several limitations to the extensibility of these observations. The atypical immunological states of the patients considered here were a clear concern. Naturally questions could be asked about the application of trends found in adults with neurosyphilis to otherwise healthy individuals in endemic settings. Further, all subjects were adults with presumably fully developed immune systems, and therefore their response may differ from children in endemic settings as well. However, none of these individuals have previously had exposure to malaria and therefore represent a sort of baseline for trends in first exposure, even if the exact values of the parameter values are not perfectly representative.

A conscious decision was made here to not limit the analysis to a single strain of *P. falciparum*. Strain-specific differences may play a large role in overall transmission dynamics [9], but often genetic information of strain diversity is limited in a particular setting. Additionally, no simple mapping between strain and pathogenicity or specific parasitaemia profiles is known to exist. Inflammatory signalling would also change in response to recent exposure [33], possibly altering the baseline relationship between fever and infections. Often in endemic settings individuals will have multiple infections simultaneously, so any mapping would also need to account for pairwise interactions or work on an assumption of independence. Handling the possibility of superinfection on this age of infection relationship would warrant further investigation in the future.

The five modalities of immunity as well as any age dependent trends will vary over time and impact all of the statistics presented here. With exposure, parasitaemia and gametocytaemia will decline; separate from that, higher parasitaemias can be tolerated before a fever develops, and transmission given a set gametocytaemia may decline. For these reasons, a static mapping from prevalence to fever rates or transmitting fraction in the absence of information on the history of exposure may be a poor representation of the epidemiological reality. Patterns of exposure (seasonality, source/sink dynamics, human travel patterns, etc) should play a large role in developing a dynamic mapping, which coincides with the understanding that malaria is a very heterogeneous disease by location. Given an understanding of the patterns of exposure, it may be possible to estimate the likely distribution of immune states in the population and take that into account for estimation of quantities of interest.

In light of these statistical relationships, if one knows an infection age distribution one can obtain estimates of fever rates and transmission potential in the absence of immunity. If a direct measure of these quantities is available, this may be able to act as a counterfactual for measuring the impact of immunity; if a model of immunity is included, one can obtain estimates of the quantities. In both cases, an infection age distribution is a crucial piece. Therefore, it places particular emphasis on the importance in determining such infection age distributions. Subsequent work is aimed to provide a model-based approach for constructing reasonable families of distributions of the age of infection given an exposure history.

Difficulty interpreting data arises in part from the extreme range of unknown previous exposure history across locations. Exposure has been measured at levels varying from no bites to more than a thousand bites by infectious mosquitoes, per person, per year; transmission efficiency, detection, and clinical manifestations of malaria depend on previous exposure and acquired immunity; exposure is often seasonal, and highly heterogeneous across individuals; and acquired immunity to malaria develops slowly, varies by exposure, protects poorly, and has poor memory [23, 38]. This prompts many studies to focus on the prevalence and outcomes in children, who can be reasonably assumed to have had little previous exposure [39]. Often patterns in children appear to more closely match trends in exposure intensity, but this leads to an identifiability problem: differences may arise due to the acquisition of immunity directly, or due to differences in the developing immune systems of children. Observed age-dependent patterns are likely a combination of both of these effects. Integrating the statistical patterns demonstrated here with mechanistic models of immunity may help to address this.

## Conclusions

Patterns relating the age of malaria infections to patency, parasitaemia, gametocytaemia, fever rates, and transmission efficiency were quantified and described. These patterns can be leveraged in population-level models of disease transmission to obtain dynamic estimates of each of these quantities. Future investigations can use these models to determine the impact of public health interventions on fever and transmission.

## Supplementary Information

**Additional file 1: R Code.**R code to read and analyse the malariatherapy data and produce the data-based figures in the manuscript.

## Data Availability

All code used during this study are at the following location: https://github.com/jmh227/Malaria-Therapy-Data-Analysis. The datasets used and/or analysed during the current study are available from the corresponding author on reasonable request.
